# Refractory flare-up of severe bronchial asthma controlled with mepolizumab due to *Pneumocystis* pneumonia: a case report

**DOI:** 10.1186/s13223-022-00678-y

**Published:** 2022-04-23

**Authors:** Kazuya Takeda, Toshiyuki Sumi, Yuta Nagahisa, Keigo Matsuura, Motoki Sekikawa, Hiroki Watanabe, Yuichi Yamada, Hirofumi Chiba

**Affiliations:** 1 Department of Pulmonary Medicine, Hakodate Goryoukaku Hospital, 38-3 Goryoukaku-Cho, Hakodate-shi, Hokkaido, 040-8611 Japan; 2 Department of Respiratory Medicine and Allergology, Sapporo Medical University School of Medicine, Sapporo, Japan

**Keywords:** Severe bronchial asthma, Mepolizumab, *Pneumocystis* pneumonia, *Pneumocystis jirovecii*

## Abstract

**Background:**

Biologics dramatically improve symptoms of severe asthma; however, various exacerbating factors may induce flare-up. *Pneumocystis *spp*.* have not been reported as a cause of asthma exacerbation during biologic use, although patients with severe asthma have high levels of antibodies against *Pneumocystis *spp.

**Case presentation:**

An 87-year-old female with severe asthma that was well-controlled with mepolizumab, who developed a steroid-resistant refractory flare-up. Chest computed tomography showed bilateral ground glass opacities, and results of polymerase chain reaction tests on induced sputum were positive for *Pneumocystis* DNA. Therefore, a diagnosis of *Pneumocystis* pneumonia was made. The clinical symptoms improved after treatment with sulfamethoxazole–trimethoprim.

**Conclusion:**

Clinicians should be aware of *Pneumocystis* pneumonia as a cause of refractory exacerbation of bronchial asthma during use of interleukin-5 inhibitors.

## Background

Although the primary treatment for severe bronchial asthma is high-dose inhaled steroids and systemic administration of steroids, symptom control has improved dramatically since the availability of biologic agents [1]. However, some patients experience exacerbations of bronchial asthma even during the use of biologic agents. The causes include allergen exposure; viral, bacterial, and fungal infections; environmental factors such as cold stimuli [2]; and the production of neutralizing antibodies to biologic agents [3], and it is difficult to control symptoms without eliminating the causes. Consequently, steroid dosage may have to be increased even when biologic agents are used.

*Pneumocystis jirovecii*, a species of the genus *Pneumocystis*, is a pathogenic microorganism that causes *Pneumocystosis* in immunocompromised hosts; the primary site of infection is the lung, and *Pneumocystis* pneumonia (PCP) is common [4]. It is noteworthy that PCP is frequently observed in patients administered steroids, immunosuppressive drugs, and biologic agents for rheumatoid arthritis and inflammatory bowel disease [5]. However, a higher percentage of children and adults with severe asthma have antibodies against *Pneumocystis *spp*.* [6, 7]. Furthermore, *Pneumocystis *spp*.*, like other allergens, are airway allergens that cause Th2-type airway inflammation [7].

Mepolizumab, an anti-interleukin (IL) -5 antibody, improves bronchial asthma symptoms by inhibiting IL-5-mediated proliferation and activation of eosinophils. However, there is no report of severe bronchial asthma complicated by *Pneumocystis* pneumonia during mepolizumab therapy, and the association is unclear.

We report a case of a patient with severe bronchial asthma that was well-controlled with mepolizumab, who subsequently developed a steroid-resistant exacerbation caused by PCP.

## Case presentation

**Fig. 1:**
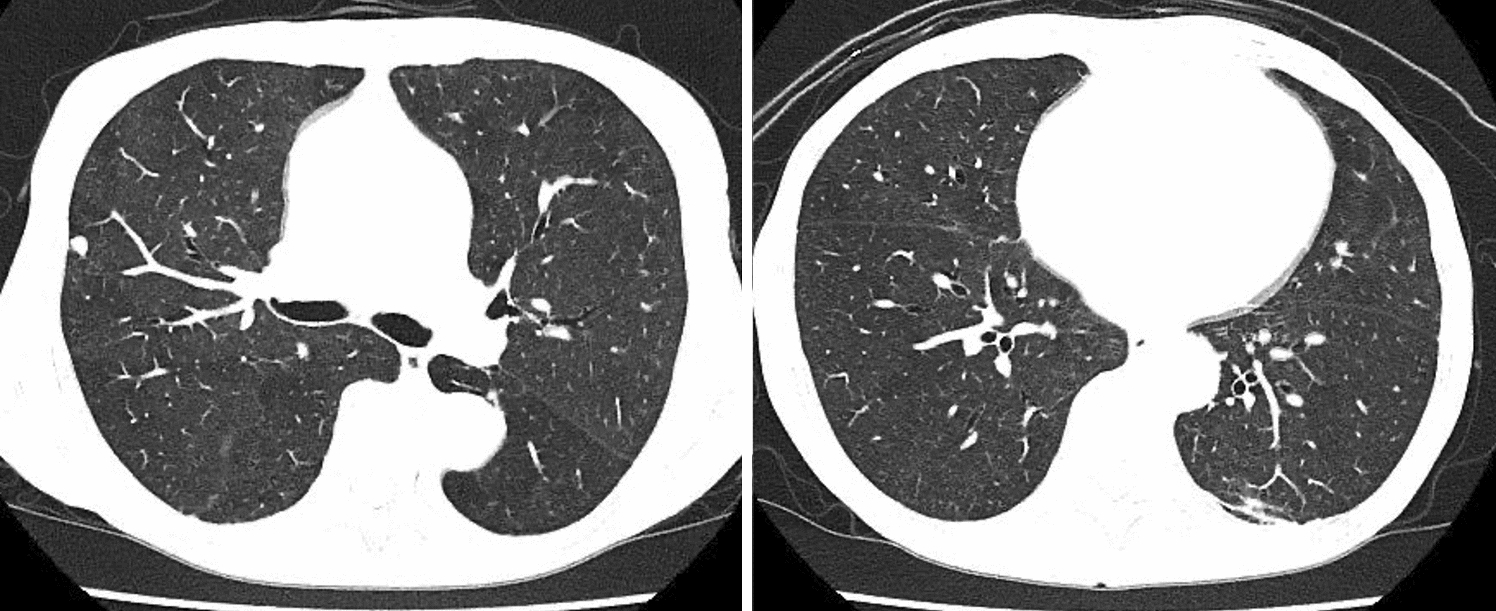
Chest computed tomography scan shows small ground-glass opacities in the bilateral lung fields

**Fig. 2:**
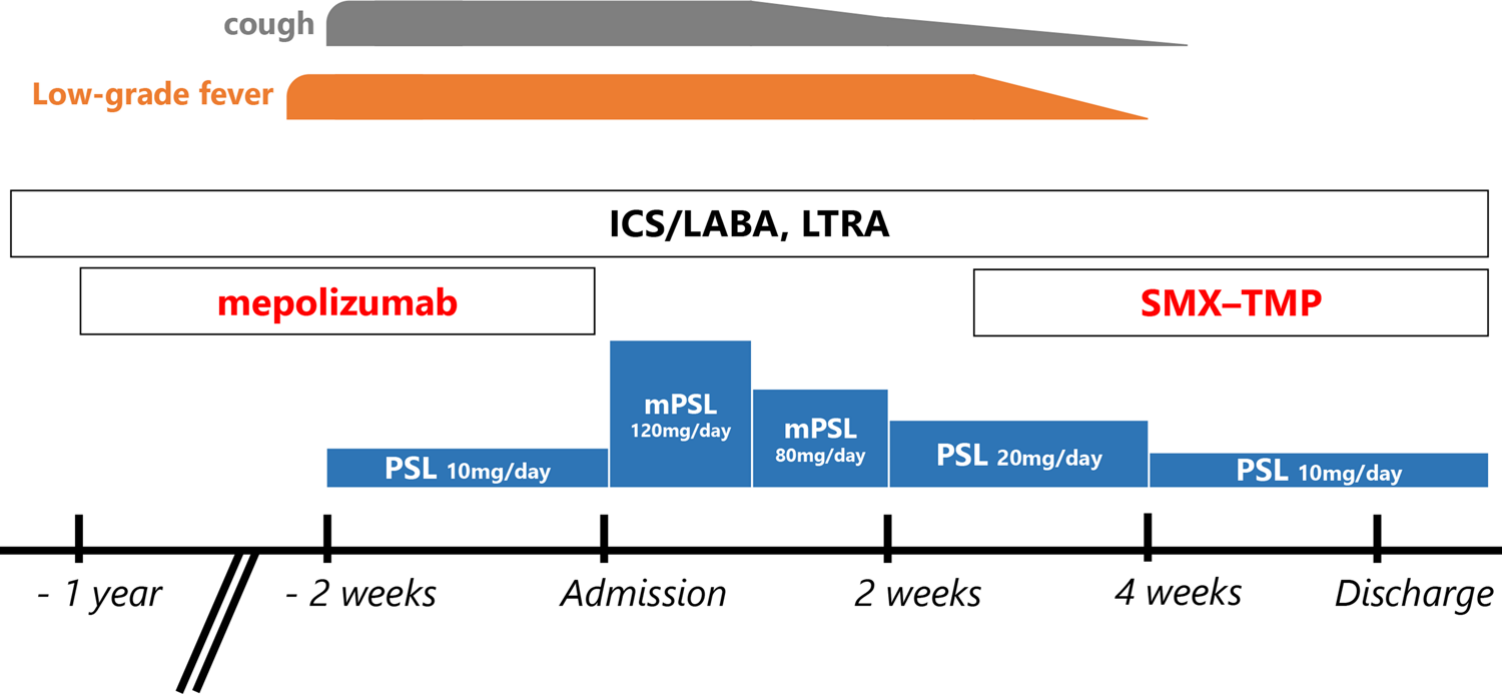
Clinical course of the patient. *ICS/LABA* inhaled corticosteroids/ long-acting beta-agonist, *LTRA* leukotriene receptor antagonist, *SMX–TMP* sulfamethoxazole–trimethoprim, *PSL* prednisolone, *mPSL* methyl prednisolone

An 87-year-old female with a history of glaucoma and bronchial asthma had been visiting the hospital for fourteen years. Her asthma phenotype was an elderly onset eosinophil-predominant, non-atopic type. She repeatedly visited the emergency room two to three times a year due to exacerbations, and each time she was treated with 20 mg of prednisolone (PSL) orally for five days. Recently, she was treated with high-dose fluticasone furoate/vilanterol and montelukast, in addition to mepolizumab, which resulted in better control and no exacerbation for one year. Thereafter, she was admitted to the hospital due to worsening bronchial asthma, mainly coughing and persistent low-grade fever of approximately 37 °C. Results of sputum Gram staining were negative, and chest radiographs showed no obvious pneumonia. She was diagnosed with exacerbation of bronchial asthma and treatment with 120 mg/day of methylprednisolone intravenously was initiated; however, the patient's cough, wheezing, and low-grade fever did not resolve. After two weeks of hospitalization, although she had no human immunodeficiency virus (HIV) infection, we considered opportunistic infection as the source of fever. Chest computed tomography showed ground glass opacities in the bilateral lung fields (Fig. 1), and a diagnosis of *Pneumocystis* pneumonia was made based on an elevated βD-glucan level of 36.4 pg/mL and positive polymerase chain reaction test results for *Pneumocystis* DNA in the induced sputum, despite negative speculum examination. Sulfamethoxazole–trimethoprim (SMX–TMP) was initiated, her fever resolved, and asthma symptoms improved in approximately one week (Fig. 2). Since then, the patient has been treated with dupilumab as a biologic agent and has not had an asthma exacerbation or used systemic steroids for more than one year.

## Discussion and conclusions

Our patient who had a steroid-resistant exacerbation of bronchial asthma during mepolizumab therapy was diagnosed with PCP based on ground-glass opacities on computed tomography and positive polymerase chain reaction test results for *Pneumocystis* DNA in the sputum. The patient was treated with SMX–TMP, which improved her symptoms. Therefore, PCP was considered as a possible cause of bronchial asthma exacerbation during anti-IL-5 antibody therapy.

There are two types of PCP: HIV-associated PCP, which occurs in patients with immunosuppression due to HIV infection, and non-HIV-associated PCP, which develops independently of HIV infection. Non-HIV-associated PCP has an acute course and is more severe than HIV-associated PCP. Moreover, the amount of pathogen at the time of infection is low and difficult to detect on speculum examination [8]. The pathogenesis of non-HIV-associated PCP is believed to be associated with an excessive immune response and correlated with inflammatory cytokines such as IL-4, IL-5, tumor necrosis factor-α, and interferon-γ [9, 10]. In mice, loss of STAT6, a transcription factor involved in Th2 cell differentiation, suppresses bronchial hypersensitivity [9]. Since Th2-type inflammatory responses are strongly associated with airway hypersensitivity symptoms in non-HIV-associated PCP, *Pneumocystis *spp*.* infection may have contributed to exacerbation and refractoriness of asthma symptoms in this case.

*Pneumocystis *spp*.* carriage rates range from 16–55% and 0–65% in patients with COPD and patients receiving immunosuppressive therapy, respectively [11]. Although there is no report on the rate of carriage in people with asthma, it is highly likely that our patient was carrying the disease, since anti-*Pneumocystis* antibodies are higher in patients with severe asthma and in older patients [7]. In this patient, PCP developed in the absence of any background HIV infection or disease indicating compromised immunity. In general, 16 mg/day of PSL for > 8 weeks increases the risk of developing PCP [12], and prophylactic administration of SMX–TMP is recommended when 20 mg/day of PSL is administered for ≥ 4 weeks [13]. In the present case, the patient had received < 5 mg/day of PSL previously and 10 mg/day of PSL for two weeks prior to admission. Therefore, no prophylactic SMX–TMP was administered because the dose and duration of PSL did not indicate the need for prophylactic SMX–TMP administration. Thus, other factors were possibly involved in the pathogenesis of *Pneumocystis* pneumonia.

First, this patient is quite elderly and it is expected that there will be some natural deviations in immune response in the lungs with aging [14]. Next, she had a high systemic glucocorticoid load according to frequent exacerbations and OCS dosing in previous years. A high OCS load is a known risk factor for severe side effects and/or complications in many clinical situations, including infectious diseases [15, 16]. And finally, the use of anti-IL-5 antibodies. IL-5 is the most potent activator of eosinophils and is produced by Th2 cells and ILC2s. Eosinophils have immunomodulatory functions in allergic diseases as well as in bacterial, fungal, and viral infections [17], and have been implicated in immunity against *Pneumocystis *spp*.* [18]. They have been shown to have killing activity against *Pneumocystis *spp*.* in a mouse model [18], suggesting that immunity against *Pneumocystis jiroveci* may be mediated by eosinophils in humans as well. In this case, the decrease in eosinophils caused by the use of an anti-IL5 antibody may have been responsible for the development of *Pneumocystis* pneumonia. Therefore, we chose dupilumab, which suppresses eosinophil activity by indirectly reducing IL-5 via Th2 cells through IL-4 inhibition, rather than direct suppression through IL-5 inhibitors, as the next biologic treatment for this patient with eosinophilic asthma.

In conclusion, *Pneumocystis *sp*.* infection should be considered as a differential diagnosis in patients with severe asthma that has been well controlled with biologic agents who develop refractory exacerbations.

## Abbreviations

PCP: *Pneumocystis* pneumonia
IL: Interleukin
PSL: Prednisolone
HIV: Human immunodeficiency virus
SMX–TMP: Sulfamethoxazole-trimethoprim
